# Dickkopf-related protein 1 is a progestomedin acting on the bovine embryo during the morula-to-blastocyst transition to program trophoblast elongation

**DOI:** 10.1038/s41598-019-48374-z

**Published:** 2019-08-14

**Authors:** Paula Tríbulo, María Belen Rabaglino, Martin Bonet Bo, Luciano de R. Carvalheira, Jeanette V. Bishop, Thomas R. Hansen, Peter J. Hansen

**Affiliations:** 1 Department of Animal Sciences, D.H. Barron Reproductive and Perinatal Biology Research Program, and Genetics Institute, University of Florida, Gainesville, FL 32611-0910 USA; 2 Instituto de Investigación en Ciencias de la Salud, CONICET, Córdoba, Argentina; 3 Embriovet S.L., A Coruña, 15165 Spain; 4 Departamento de Clínica e Cirugia Veterinárias, Escola de Veterinária, Universidade Federal de Minas Gerais, Belo Horizonte, Minas Gerais Brazil; 5 Department of Biomedical Sciences, Colorado State University, Fort Collins, CO 80523-1683 USA

**Keywords:** Reproductive biology, Embryology

## Abstract

Progesterone regulates the endometrium to support pregnancy establishment and maintenance. In the ruminant, one action of progesterone early in pregnancy is to alter embryonic development and hasten the process of trophoblast elongation around day 14–15 of pregnancy, which is required for maternal recognition of pregnancy. Here we demonstrate that the WNT antagonist DKK1, whose expression is increased by progesterone treatment, can act on the bovine embryo during day 5 to 7.5 of development (the morula to blastocyst stage) to promote embryonic elongation on day 15 of pregnancy. Embryos were produced *in vitro* and exposed to 0 or 100 ng/ml recombinant human DKK1 from day 5 to 7.5 of culture. Blastocysts were transferred into synchronized recipient cows on day 7.5 (n = 23 for control and 17 for DKK1). On day 15, cows were slaughtered and embryos recovered by flushing the uterus. Embryo recovery was n = 11 for controls (48% recovery) and n = 11 for DKK1 (65% recovery). Except for two DKK1 embryos, all embryos were filamentous. Treatment with DKK1 increased (P = 0.007) the length of filamentous embryos from 43.9 mm to 117.4 mm and the intrauterine content of the maternal recognition of pregnancy signal IFNT (P = 0.01) from 4.9 µg to 16.6 µg. Determination of differentially expressed genes (DEG), using the R environment, revealed 473 DEG at p < 0.05 but none at FDR < 0.05, suggesting that DKK1 did not strongly modify the embryo transcriptome at the time it was measured. However, samples clustered apart in a multidimensional scaling analyisis. Weighted gene co-expression analysis of the transcriptome of filamentous embryos revealed a subset of genes that were related to embryo length, with identification of a significant module of genes in the DKK1 group only. Thus, several of the differences between DKK1 and control groups in gene expression were due to differences in embryo length. In conclusion, DKK1 can act on the morula-to-blastocyst stage embryo to modify subsequent trophoblast elongation. Higher pregnancy rates associated with transfer of DKK1-treated embryos may be due in part to enhancements of trophoblast growth and antiluteolytic signaling through IFNT secretion. Given that progesterone can regulate both timing of trophoblast elongation and DKK1 expression, DKK1 may be a mediator of progesterone effects on embryonic development.

## Introduction

The uterine environment plays a critical role in embryonic development and survival. In cattle, it has been estimated that only ~60 to 70% of females are capable of supporting embryonic development to term^1^. In ruminants, as in other species, endometrial function is under the regulation of ovarian steroids^2–5^. One action of progesterone is to modify the uterine environment to facilitate elongation of the trophoblast around day 14–17 of pregnancy. Treatment with progesterone early after ovulation increases trophoblast elongation and IFNT secretion by the embryo^6–10^, whereas experimental reduction in concentrations of progesterone resulted in smaller embryos at day 14^3^.

Rapid elongation of the trophoblast to form a filamentous, free floating embryo around the time of gastrulation is a characteristic of preimplantation development in ungulate species such as the ruminants and the pig^11^. Elongation generates sufficient surface area of the trophoblast for nutrient uptake and preparation for placentation and also is accompanied by changes in trophoblast gene expression that generate antiluteolytic signals to the mother to prevent luteolysis^12–14^. Trophoblast elongation can occur in an explosive fashion. The bovine embryo increases from 2–5 mm in length between days 13 and 14 of development to 60–150 mm in length on day 16^15,16^. Elongation involves cell proliferation and cellular rearrangement and is accompanied by changes in embryo gene expression^11,14,17^. In the ruminant, expression of the antiluteolytic signal IFNT is massively upregulated coincident with trophoblast elongation^18^. *IFNT* expression and IFNT secretion by the elongating embryo is proportional to embryo length^19,20^.

Effects of progesterone on elongation of the bovine embryo are mediated by changes in endometrial function rather than by direct effects of progesterone on the embryo because culture of embryos with progesterone has no effects on subsequent elongation^9^. Local mediators of the actions of progesterone on target tissues have been termed progestomedins^21^. For the most part, these molecules remain unidentified. One possible mediator of the effects of progesterone on embryonic elongation in the ruminant is DKK1, an endogenous modulator of WNT signaling produced by the endometrium^22^. Addition of DKK1 to culture medium improves the ability of bovine embryos to establish and maintain pregnancy after transfer^23^ and affects birthweight of offspring^24^. Endometrial expression of *DKK1* is induced by progesterone in the cow^3^, sheep^25^ and human^26,27^. Endometrial expression of *DKK1* in cattle has been reported to be more highly expressed in endometrium of fertile animals than endometrium of infertile animals^28,29^.

Here, it was hypothesized that DKK1 is a progestomedin which modifies embryonic development and IFNT secretion. The objective of this study was to determine the effects of DKK1 treatment to embryos at days 5–7 of culture on embryo length, IFNT accumulation in the uterus and embryonic transcriptome at day 15.

## Methods

All procedures involving cows were approved by the Animal Care and Use Committee of the University of Florida and all methods were performed in accordance with the relevant guidelines and regulations.

### Embryo production

Embryos were produced *in vitro* using Holstein oocytes harvested from slaughterhouse ovaries [purchased from Simplot (Emmett, ID, USA) for replicate 1 and by Desoto BioScience (Seymour, TN, USA) for replicate 2 and 3] and X-sorted spermatozoa from a single Holstein sire (Mr Rubicon Dynasty, Sexing Technologies Genetics, Navasota, TX, USA). Procedures for *in vitro* oocyte maturation, *in vitro* fertilization and embryo culture were described previously^30–33^ with the exception that culture took place in a benchtop incubator (Minc^TM^, Cook Medical, Bloomington, IN, USA) at 38.5 °C in a humidified atmosphere of 5% (v/v) O_2_ and 5% (v/v) CO_2_ with the balance N_2_ supplied as premixed gas. Beginning at day 5 after fertilization, embryos were cultured with either 0 or 100 ng/ml human recombinant DKK1 (R&D Systems, Minneapolis, MN, USA) following procedures described previously^33,34^. Dose was chosen because it was effective in increasing pregnancy rates^33^.

### Recipients

Non-lactating Holstein cows at the University of Florida Dairy Research Unit (Hague, FL, USA) were used as embryo recipients. Animals were synchronized for timed-embryo transfer using the Ovsynch protocol^35^ consisting of administration of 100 µg gonadotropin releasing hormone (GnRH) (Cystorelin^®^, Merial, Lyon, France) on day −10, 25 mg prostaglandin F_2α_ (Lutalyse^®^, Zoetis, Parsippany-Troy Hills, NJ, USA) on day −3, followed by 100 µg GnRH (Cystorelin^®^) on day −1. Day of expected ovulation was considered day 0 and the day of transfer was day 7.

### Embryo transfer

Grade 1 blastocyst and expanded-blastocyst stage embryos^36^ were harvested from culture on day 7.5 after insemination. Selected embryos were placed into holding medium (BO-Transfer, IVF Bioscience, Falmouth, UK) and loaded individually into 0.25 ml French straws. Loaded straws were placed into a portable incubator (BioTherm, CryoLogic, Blackburn, Victoria, Australia) set at 38.5 °C and transported to the farm for transfer to recipients.

On day 7 after expected ovulation, the ovaries of all cows were examined by transrectal ultrasonography using an 8–5 MHz linear-array probe (Ibex^®^ Pro, E.I. Medical Imaging^®^, Loveland, CO, USA) to determine the presence or absence of a corpus luteum. Only cows which had a visible corpus luteum on day 7 were selected as a recipient. A total of 40 cows were selected based on this criterion. Selected recipients were given a dose of 5 ml of 2% (w/v) lidocaine hydrochloride (Aspen Veterinary Resources, Ltd., Liberty, MO, USA) in the sacro-coccygeal space or first intercoccygeal junction to induce caudal epidural anesthesia. A single blastocyst was transferred to the uterine horn ipsilateral to the side of the ovary with a corpus luteum. A total of 23 control and 17 DKK1-treated embryos were transferred.

### Recovery of uterine flushings and embryos

Recipients were slaughtered on day 15 after expected ovulation by captive bolt stunning and exsanguination. Reproductive tracts were obtained immediately thereafter and placed on ice. Processing of all organs was completed within a maximum of 3 h after slaughter of the first cow.

Uteri were flushed with 50 ml Dulbeccos’s phosphate-buffered saline (DPBS). A hemostatic clamp was placed over the cervix and flushing fluid was introduced into the uterine horn contralateral to the side of ovulation and massaged through the uterus to the uterine horn ipsilateral to the side of ovulation. The end of the uterine horn ipsilateral to the side of ovulation near to the uterotubal junction was opened by dissection and flushing fluid was propelled by massage through the opening and harvested in a petri-dish (Falcon^®^ Integrid^TM^ dishes, Corning, NY, USA). The procedure was repeated three times unless an embryo was observed first. The fluid collected from the first flushing was transferred to a 50 ml tube (Sarstedt AG, Nümbrecht, Germany) and kept on ice. The embryo, if present, was removed with forceps and the flushings were centrifuged at 3000 × g for 15 min at 4 °C. The supernatant fraction was obtained and stored at −20 °C.

### Characterization of embryo morphology

Embryo length was measured using a ruler, with the embryo laid out along a line of the grid plate. When embryos were longer than the plate, they were arranged in parallel lines while folding the embryo. In addition, embryos were classified based on morphology and length as either tubular (5 to 15 mm) or filamentous (≥15 mm). After measurements were recorded, embryos were snap frozen in liquid nitrogen (−196 °C) and then stored at −80 °C.

### Concentration of IFNT in uterine flushings

Glycosylated recombinant bovine (rb)IFNT was purified from cultures of human HEK cells that were transformed with bovine IFNT cDNA (bTP509) and used to generate polyclonal antibodies in goats (#51; 3.5 μg/ml) and in rabbits (#5670; 9.6 μg/ml) (J.V. Bishop and T.R. Hansen, unpublished in collaboration with a biopharma company). These antibodies were used as capture and biotinylated detector antibodies, respectively, in a sandwich enzyme linked immunosorbent assay (ELISA). The ELISA had a range of detection of 7.8 to 500 pg/ml and the limit of detection was 31.25 pg/ml. The intra-assay coefficient of variation was 0–1.4% for high (500 pg/ml), 0–3.9% for medium (100 pg/ml) and 0.9–2.2% for low (20 pg/ml) concentration rbIFNT controls. The inter-assay coefficient of variation was 1.1%, 1.6% and 1.8% for high, medium and low concentration controls, respectively. The ELISA specifically detects IFNT and does not cross-react with IFNω, IFNα/β or IFNγ. All samples of uterine flushings were analyzed blind. Samples were assayed undiluted or at dilutions of 1:10, 1:100, 1:1,000, 1:5,000 or 1:10,000 (w/v) to detect IFNT in the linear range of the assay.

Total amount of IFNT in the uterine lumen was calculated by multiplying the concentration of IFNT by the volume of DPBS used for the first flushing. Concentrations under the detection limit of the assay were considered to be 31.25 pg/ml (detection limit of the assay) for statistical purposes.

### Statistical analysis of data on embryo recovery, embryo size and IFNT content

Binary responses were analyzed by multivariable logistic regression using the GLIMMIX procedure of SAS version 9.4 (SAS Institute Inc., Cary, NC) fitting binomial distribution. Response variables were the proportion of putative or cleaved zygotes that developed to the blastocyst stage at day 7.5 after insemination and the proportion of transferred blastocysts that were recovered on day 15. The statistical model included the fixed effect of treatment and random effect of replicate.

Treatment effect on filamentous embryo length and IFNT content in the uterus was determined by analysis of variance using the MIXED procedure of SAS including the fixed effects of treatment and random effect of replicate. Embryo length from one embryo from the control group was not obtained because the embryo was recovered in several pieces; data from this embryo was excluded. Data for embryo length and IFNT content were log transformed to meet the assumptions of normal distribution.

### RNA extraction and sequencing

Gene expression analysis was performed using RNA from filamentous embryos (n = 11 for control and n = 9 for DKK1). Embryos were thawed and subjected to RNA extraction using the Qiagen RNeasy Mini kit (Qiagen, Valenciam, CA, USA) following manufacturer instructions including DNase treatment. RNA concentration was determined on Qubit® 2.0 Fluorometer (ThermoFisher/Invitrogen, Grand Island, NY, USA). The RNA quality was assessed using the Agilent 2100 Bioanalyzer (Agilent Technologies, Inc.). All samples met the criteria for library construction (total RNA with 28S/18S > 1 and RNA integrity number ≥7).

RNA library construction was performed at the Interdisciplinary Center for Biotechnology Research (ICBR) Gene Expression & Genotyping Core, University of Florida. A total of 25 ng of total RNA was used for mRNA isolation with the NEBNext Poly(A) mRNA Magnetic Isolation Module (New England Biolabs) and RNA library construction followed with NEBNext® Ultra™ Directional RNA Library Prep Kit (New England Biolabs) according to the manufacturer’s user guide. Briefly, RNA was fragmented followed by first strand cDNA synthesis using reverse transcriptase and oligo-dT primers. Synthesis of double strand cDNA was performed, followed by end-repair and adaptor ligation. At this point, Illumina adaptors were ligated to the sample. Finally, the library was enriched (each library had a unique barcode) by 10 cycles of amplification, and purified by Agencourt^®^ AMPure beads (Beckman Coulter Life Sciences, Indianapolis, IN, USA). Eight barcoded libraries were sized on the Bioanalyzer, quantitated by Qubit dsDNA HS assay (Thermo Scientific). Finally, equimolar amounts of 20 individual libraries were pooled and sequenced by Illumina HiSeq 3000 2 × 100 cycles run for total of 3 runs (Illumina Inc., San Diego, CA, USA)

### Processing and analysis of RNA-Seq data

Raw paired-end reads generated from the Illumina HiSeq. 3000 sequencing platform were cleaned up with the Cutadapt program^37^. The partial adaptors and potential sequencing errors introduced during sequencing or library preparation were trimmed off and reads with a quality and read length <40 bases were excluded from RNA-seq analysis. The transcripts of the *Bos taurus* genome (57,846 sequences) retrieved from the NCBI genome database were used as reference sequences for RNA-seq analysis. All processed paired-end reads of each sample were individually mapped to the reference sequences using bowtie2 (v. 2.2.3) with a ‘3 mismatches within a read’ allowance^38^. The samtools and scripts developed in house at the ICBR were used to detect the potential PCR duplicates and to choose uniquely mapped reads for gene expression analysis. The gene expression levels were assessed by counting the number of mapped reads for each gene symbol^39^. Selected statistics on raw reads and successful mapping are shown in Supplementary Dataset 1 Tab 1 (Table S1). Except for three samples, the mapping percent ranged from 45.1 to 50.7%. The exception was for 3 of the 9 samples from the DKK1 group. Two of these samples were excluded from subsequent analysis because of poor mapping (24.96 and 0.45% mapped reads, respectively), and one (38.38% mapped reads) was excluded because it was identified as an outlier during statistical analysis. Thus, the final number of samples was 11 for control and 6 for DKK1.

The RNASeq data generated in the study have been deposited at Gene Expression Omnibus (GEO) with the accession number GSE126680.

### Statistical analysis of RNAseq data

The following procedures were performed with the edgeR package for the R software^40^. Genes with low expression counts [less than 1 count per million (CPM) in 6 or more samples across treatment], were filtered out before normalization. With this criterion, 7,897 transcripts were filtered out and 10,006 were retained for further analysis. The normalization method applied was TMM (weighted trimmed mean of M-values)^41^. Normalization factors for all samples had a mean of 1, with a minimum of 0.92 and a maximum of 1.09.

To visualize the level of similarity of the samples, multidimensional scaling plot of distance between digital gene expression profiles (MDS) for the top 500 most variable genes, were generated using plotMDS function of edgeR package in R.

A robust estimate of the negative binomial dispersion parameter for each gene, using observation weights, was applied to avoid outlier genes, which can lead to the detection of false positives. These observation weights were used later for estimating regression parameters. The method uses an iterative procedure where weights are calculated from residuals and estimates are made after re-weighting^42^. Finally, a negative binomial generalized log-linear model was fit to conduct gene-wise statistical tests for the coefficient contrast^43^. The matrix of contrast was built based on the comparison between groups (DKK1 vs control). Differentially expressed genes (DEG) were determined through one-way analysis of variance.

An additional statistical analysis was made with a subset of samples to discern the effect of DKK1 on gene expression independent of effect on embryo length. The four shortest (short control) and four longest embryos (long control) from the control group and four embryos from the DKK1 group whose length was similar to those in the subset of long embryos were included in this analysis. The four DKK1 embryos were chosen to ensure that their length fell within the range of lengths of the long control group. Length averaged 30.5 mm (range: 15 to 47 mm) for short control, 128.8 mm (range: 90 to 180 mm) for long control and 121.8 mm (range: 92 to 160 mm) for the DKK1 group. For this analysis, 7,794 transcripts with low expression counts (less than 1 CPM in 4 or more samples across treatments) were filtered out before normalization and 10,109 were retained for further analysis. Differentially expressed genes (DEG) were determined through one-way analysis of variance, applying an F-test to identify DEG among the groups. For statistical analysis, comparisons were DKK1 vs. short control, DKK1 vs. long control, and long control vs short control. The method combines the pairwise comparisons into a single F-statistic and p-value. DEG were defined as those with a p-value < 0.05 and a fold change (FC) > 1.5 for each pairwise comparison.

Additionally, a gene co-expression analysis was performed for describing the correlation patterns among genes across treatments. Samples from each group (DKK1 or control) were subjected to weighted gene co-expression network analysis (WGCNA)^44^, with the WGCNA package for R software. Gene counts were normalized by CPM and log2 transformed. Genes with low counts (less than 10 CPM in 90% of the samples for each group) were filtered out. The number of genes remaining were 4858 in the DKK1 group and 4768 in the control group. The automatic method was employed for block-wise signed network construction and module detection. The co-expression similarity was raised to a soft thresholding power (β) of 18 to calculate adjacency. Parameters were adjusted to generate the same number of modules for each group [10 modules of co-expressed genes plus 1 module (coded grey) of outsider genes]. The resulting modules for each network were related with the embryo length to identify modules, or clusters of co-expressed genes significantly correlated with embryo length. Module membership (MM) is a measure of the degree of correlation of an individual gene with other genes in the module (the higher the MM, the more connected it is to other genes in the module). Gene significance (GS) is the correlation of each gene within a module with embryo length. Specific subsets of genes identified using the co-expression analysis were uploaded to Ingenuity Pathway Analysis (IPA; version 01.13, Qiagen) software for clustering of genes into common molecular and cellular function terms, for clustering into physiological system development and function terms, and to identify upstream regulators that were either transcriptional regulators or cell signaling molecules (growth factors, cytokines, G-coupled receptors and ligand-dependent nuclear receptors). Only terms or upstream regulators in which the P value was 0.05 or less are reported.

## Results

### Characteristics of embryo development and survival to day 15

Addition of DKK1 to culture did not affect the proportion of either zygotes or cleaved embryos that developed to the blastocyst stage (16.0 ± 2.4 vs. 14.6 ± 2.3, P = 0.63 and 22.7 ± 3.1 vs. 20.0 ± 2.9, P = 0.52).

**Figure 1:**
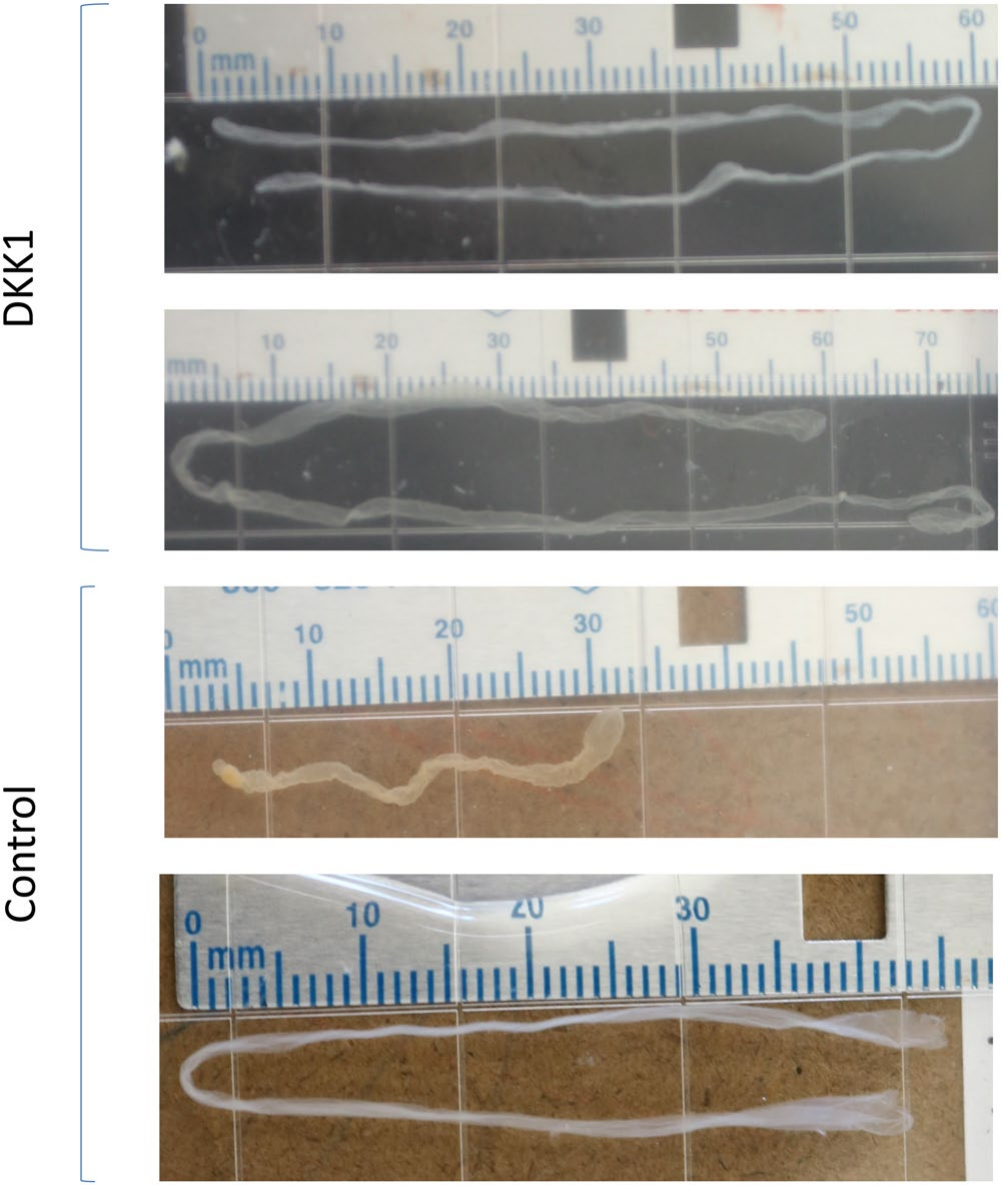
Representative images of recovered conceptuses.

Following transfer to recipients and retrieval at day 15, embryos were recovered from 11 of 23 recipients for controls (48% recovery) and from 11 of 17 recipients for DKK1 (65% recovery). Representative images of embryos are shown in Fig. 1. With two exceptions, all recovered embryos were filamentous. One DKK1 embryo was tubular and one was only recovered as a single, incomplete piece so that morphology could not be determined. One filamentous embryo in the control group was recovered in several fragments so that length could not be estimated. Examination of IFNT in uterine flushings was used to determine the accuracy of embryo recovery as a measure of pregnancy status. All of the cows in which an embryo was recovered had detectable amounts of IFNT in uterine flushings. In addition, one cow in the DKK1 group in which an embryo was not recovered had a total of 600 ng IFNT. None of the other cows in which an embryo was not recovered had detectable IFNT in uterine flushings.

**Figure 2:**
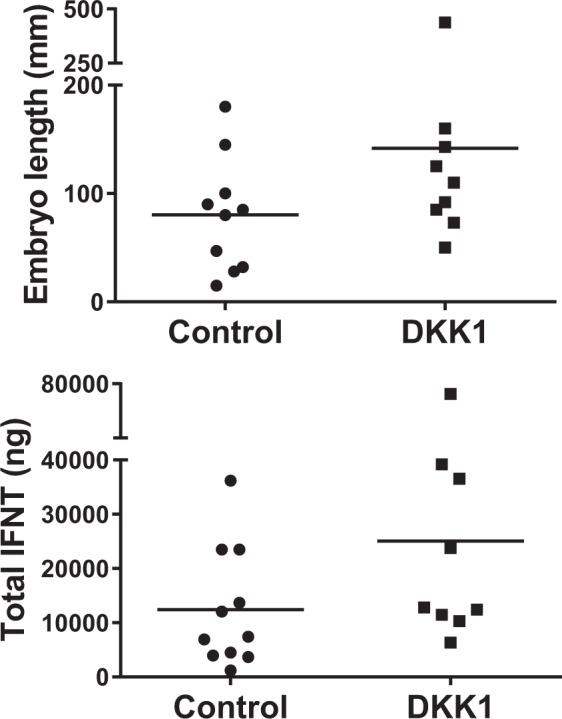
Individual values of length of recovered filamentous embryos (top) and total IFNT in pregnant cows (bottom). The horizontal bars represent the aritmetric mean for each treatment. Both embryo length and total IFNT were increased by exposure of embryos to DKK1 (P = 0.007 and P = 0.01, respectively).

As shown in Fig. 2, DKK1 increased (P = 0.007) the length of filamentous embryos and the amount of immunoreactive IFNT accumulated in uterine flushing of cows with a filamentous embryo (P = 0.01).

### Effect of DKK1 on the transcriptome of day 15 filamentous embryos

**Figure 3:**
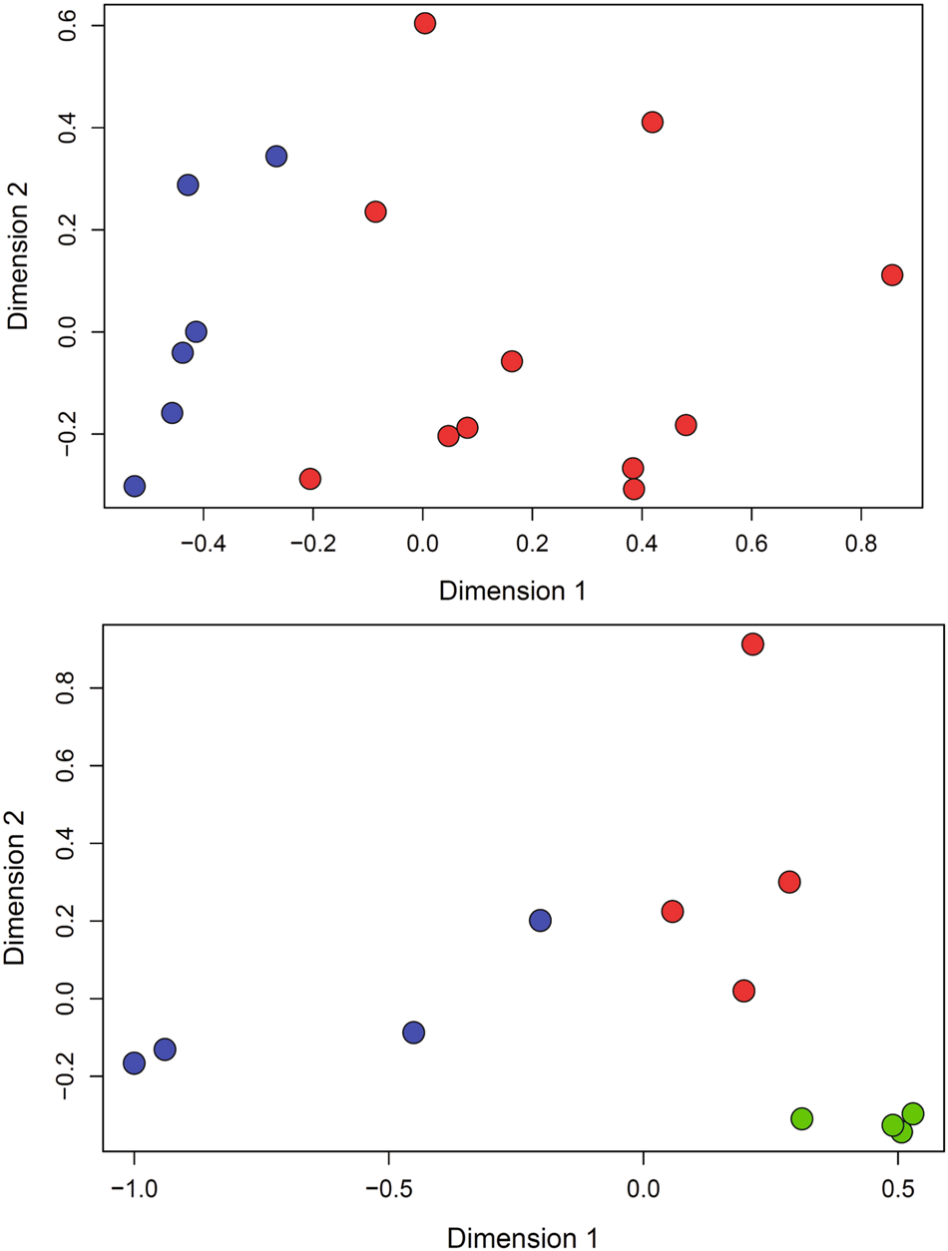
Multidimensional scaling analysis for transcriptomes of individual samples of filamentous embryos treated with DKK1 (blue) or control embryos (red) (top) and for a subset of filamentous embryos representing short control embryos (blue), long control embryos (red) or DKK1 embryos of the same length as long control embryos (green) (bottom).

A total of 17,903 genes were expressed in at least one day 15 filamentous embryo (Table S2 in Supplementary Dataset 1 Tab 2). Genes with low expression counts [less than 1 CPM in 6 or more samples across treatments], were filtered out before normalization. With this criterion, 7,897 transcripts were filtered out and 10,006 were retained for further analysis. The MDS analysis showed that embryos exposed to DKK1 clustered together, and differed from those in the control group, mainly in the first dimension, which accounts for the highest variability between samples (Fig. 3). A total of 473 genes were differentially expressed between DKK1 and control embryos (P < 0.05), with 273 upregulated by DKK1 and 200 downregulated (Supplementary Dataset 1 Tab 2). For most DEG, the difference in expression between groups was small. Only 182 DEG genes had a fold change >1.5 (113 upregulated and 69 downregulated) and 48 with a fold-change >2.0 (26 upregulated and 22 downregulated). Correction for a false discovery rate (FDR) of ≤0.1 resulted in no DEG.

We investigated the presence of markers for bovine trophoblast, mesoderm and endoderm^45^ among the 182 genes affected by DKK1. There was only one marker for endoderm (*AQP11*), eight markers for mesoderm (*APEH*, *HMGA1*, *L3HYPDH*, *MAST1*, *MEG3*, *NPPB*, *RPL26L1*, *SF3B2*), and 30 markers for trophoblast (*ARHGAP1*, *AZI2*, *BEX2*, *CD99L2*, *CDKL5*, *CDKN1A*, *DHRS9*, *DNASE1L3*, *FAM234A*, *FGD4*, *GNAI1*, *KCTD18*, *MOV10*, *OPLAH*, *PAG12*, *PLA2G7*, *PTK2B*, *PUM1*, *RAB32*, *RNF122*, *RUFY3*, *SERPING1*, *SLC4A5*, *TADA2A*, *THY1*, *TRIM25*,*TSPAN1*, *UTP11*, *ZKSCAN5*, *ZWILCH*). Despite the difference in accumulation of IFNT in the uterus between groups, expression of IFNT was not affected by treatment (21658.9 vs 17145.2 counts per million base pairs for DKK1 and control, respectively).

### Changes in the relationship between embryo length and gene expression caused by DKK1

Elongation of the bovine embryo at day 15 is associated with wide scale changes in gene expression^18^. Initially an analysis was performed to to determine effects of DKK1 on gene expression at day 15 due to changes in embryo length vs those independent of length. The analysis included comparison of gene expression of short control embryos, long control embryos and a subset of DKK1 embryos having similar lengths to the long control embryos. MDS analysis revealed that the three groups of embryos clustered into three groups (Fig. 3). There were 698 genes that differed between groups (Table S3 in Supplementary Dataset 1 Tab 3) but none were significant after correcting for FDR.

Subsequently, gene co-expression analysis was used as a tool to identify networks of co-expressed genes correlated with embryonic length. Results are shown in Table 1 and Supplementary Dataset 1 Tab 4 (DKK1; Table S4) and 5 (control; Table S5). In the DKK1 group, there were 10 modules of genes identified but only one (designated as the green module) was significantly correlated with length (0.81, P-value = 0.05). A total of 377 of the genes in the green module were annotated by IPA. The top molecular and cellular functions in which these genes were represented (based on P value) were cell death and survival (160 genes), gene expression (114 genes), and RNA post-transcriptional modification (32 genes). The top physiological system development and function terms in which genes clustered were embryonic development (112 genes) and organismal development (120 genes). The most significant upstream regulators that were transcriptional regulators (all P < 0.05): TP53 (71 genes), NUPR1 (26 genes), HNF4A (64 genes) and MYCN (15 genes). The most significant upstream regulators that were cell signaling molecules: ESR1 (40 genes), IL15 (18 genes), CSF2 (17 genes), and HGF (17 genes).

**Table 1:** Results of gene co-expression analysis for transcriptome of embryos at day 15 of development either treated with DKK1 or control conditions during days 5 to 7 of culture.

Module name	Module size (number of genes)	Correlation	P-value
**Control group**			
Modules negatively correlated with embryo length			
Green	365	−0.45	0.2
Yellow	376	−0.4	0.2
Black	312	−0.35	0.3
Magenta	203	−0.24	0.5
Brown	581	−0.097	0.8
Turquoise	1491	−0.03	0.9
Modules positively correlated with embryo length			
Purple	75	0.047	0.9
Pink	261	0.083	0.8
Red	353	0.094	0.8
Blue	721	0.25	0.5
Module with outsider genes			
Grey	30		
**DKK1 group**			
Modules negatively correlated with embryo length			
Black	110	−0.69	0.1
Purple	58	−0.56	0.2
Blue	988	−0.24	0.6
Brown	960	−0.13	0.8
Red	132	−0.07	0.9
Modules positively correlated with embryo length			
Pink	68	0.094	0.9
Yellow	955	0.12	0.8
Magenta	62	0.14	0.8
Turquoise	1126	0.46	0.4
Green	395	0.81	0.05
Module with outsider genes			
Grey	4		

In the green module, 36% of the individual genes had a correlation with embryo length with p < 0.1 for GS (143 out of 395 genes) and 91 had P < 0.05. All of these correlations were positive. Thus, longer embryos have higher expression of the genes in this module than shorter embryos. In contrast, none of the modules in the control group were significantly associated with embryo length. Genes in the green module for the DKK1 group that had a GS of P < 0.05 were analyzed by IPA. Of the 89 genes annotated, the top molecular and cellular functions in which these genes clustered (based on P values) were molecular transport (24 genes), RNA trafficking (7 genes), DNA replication, recombination and repair (19 genes) and cell death and survival (41 genes). The top physiological system development and function terms in which genes clustered were cardiovascular system development and function (15 genes) and organismal development (15 genes). The most significant upstream regulators that were transcriptional regulators were TP53 (18 genes), VAX2 (1 gene) and HNF4A (16 genes). The most significant upstream regulators that were cell signaling molecules were KITLG (6 genes), TGFB1 (17 molecules) and TNFA (13 molecules).

Among all genes in the DKK1 group, 3.3% (159/4858) were significantly (P < 0.05) correlated with embryo length, with 133 genes being positively correlated and 26 being negatively correlated (Table S1 in Supplementary Dataset 1 Tab 4). For all genes in the control group, 3.0% (145/4768) were significantly (P < 0.05) correlated with embryo length, with 51 genes being positively correlated and 94 being negatively correlated (Table S5 in Supplementary Dataset 1 Tab 5). Only 10 of the genes that were significantly correlated with embryo length in the DKK1 group were also significantly correlated and in the same direction in the control group (positive correlation: *ANXA11*, *C3H1orf123*, *DDB1*, *IMPA2*, *LRRC42*, and *PBDC1;* negative correlation: *ABCD1*, *MPLKIP* and *RFNG)*.

## Discussion

Here, it is demonstrated that the WNT antagonist, DKK1, can regulate development of the bovine embryo during the morula and blastocyst stages at days 5 to 7 of development to promote trophoblast elongation and IFNT secretion by the filamentous embryo at day 15 of development. Thus, DKK1 can program critical events during the morula to blastocyst transition to regulate subsequent trophoblast elongation.

The idea that trophoblast elongation is under the control of events taking place earlier in gestation is not new. Experiments involving transfer of embryos into recipients have demonstrated that events a week before onset of trophoblast elongation can affect the timing or extent of the elongation process. Treatment of *in vitro* produced bovine embryos with CSF2 from day 5 to 7 of development altered characteristics of elongation at day 15 in a sex-dependent manner^46^. CSF2 decreased embryo length and intrauterine accumulation of IFNT for female embryos and increased these measurements for male embryos. Shorten *et al*.^20^ found that embryo size at day 7 of development (day of transfer) was associated positively with trophectoderm length and IFNT expression at day 15.

The most novel insight from the current study is the implication that DKK1 is likely to be a progestomedin that mediates the well-described actions of progesterone to promote trophoblast elongation in cattle^3,6–10^. Endometrial expression of *DKK1* is increased by progesterone not only in the cow^3^ but also in the sheep^25^ and human^26,27^. Perhaps, DKK1’s role as a progestomedin is not limited to regulation of trophoblast elongation in ruminants but the protein may function as a mediator of progesterone action on the endometrium in multiple mammalian species.

The present findings also provide additional evidence for the importance of the WNT signaling pathway for early pregnancy. Various WNT signaling genes have been implicated in preimplantation development, endometrial morphogenesis, implantation and trophoblast function^47–49^. In the cow, overactivation of β-catenin dependent WNT signaling can compromise the proportion of embryos developing to the blastocyst stage; DKK1 can counter this effect^33,34^. Moreover, blastocysts produced in the presence of DKK1 were better able to establish and maintain pregnancy after transfer into recipients than control embryos^23^. It can be inferred from these findings that DKK1 is an important endometrial determinant of embryonic development. Further evidence for this idea comes from studies showing that transcripts for *DKK1* are more abundant in endometrium of heifers identified as inherently fertile than in endometrium of heifers identified as inherently infertile^29^. Similarly, endometrial expression of *DKK1* was lower for lactating cows, characteristically of low fertility, than for non-lactating cows^28^. Later in pregnancy, *DKK1* is one of the genes whose transcript abundance is increased in endometrium of the cow at day 18 of pregnancy, coincident with impending placentation and maximum IFNT secretion^50^.

Given the importance of IFNT for establishment of pregnancy in cattle, it is likely that the increase in IFNT secretion caused by DKK1 is one reason why embryos treated with DKK1 had higher pregnancy rate after transfer to recipients than control embryos^23^. The DKK1-induced increase in IFNT secretion was caused by the increase in trophoblast length associated with DKK1 treatment and not by increased steady-state amounts of *IFNT* mRNA per cell. This is because there was no effect of DKK1 on *IFNT* expression as determined by RNA Seq. Many of the genes whose expression were changed by DKK1 also reflect the increase in embryo length, i.e., a subset of 307 genes regulated by DKK1 was also differentially expressed between long and short embryos in the control group.

There were differences in the transcriptome between embryos treated with DKK1 and control embryos as indicated by MDS analysis (Fig. 3). These differences were for the most part small in magnitude. Many were probably related to differences in embryo length between embryos treated with DKK1 and control embryos. The fact that there was a module of genes whose expression was positively correlated with embryo length for the DKK1 group but not for the control group is a reflection of the longer embryos in the DKK1 group. Moreover, there were more genes whose expression was positively related to embryo length for the DKK1 group than for the control group, despite the fact that the power of the analysis was lower for the DKK1 group (because of smaller sample size). It is likely, however, that there are differences in gene expression between DKK1 and control embryos independent of differences in embryo length because a subset of control embryos of the same length as a subset of DKK1 embryos formed a distinct cluster from the DKK1 group (Fig. 3).

There is other evidence that DKK1 changes aspects of conceptus function besides those related to trophoblast elongation. There is a report that calves derived from DKK1-treated embryos had lower birth weight than calves from embryos not treated with DKK1^24^. The period from hatching to placentation takes around two weeks in ruminants and it is at this time when formation of the embryonic disc and gastrulation occur^11,51^. Perhaps DKK1 alters the pattern of gene expression of the embryo in a way that affects subsequent fetal development. Most of the genes examined in the current experiment are derived from trophoblast because this is the predominant tissue in the Day 15 embryo. Future experiments examining actions of DKK1 on gene expression in the embryonic disc could provide further insights into how DKK1 may alter gastrulation and subsequent developmental events.

WNT signaling has been identified as a pathway associated with uterine capacity for pregnancy and fertility in beef cattle^29^, as well as a candidate mechanism for maternal regulation of embryonic development^52,53^. It is likely that DKK1 is not the only WNT signaling molecule important for fertility. Another WNT ligand, WNT7A, which is produced in the endometrium of the cow^22^, can increase competence of *in vitro* produced embryos to develop to the blastocyst stage^34,54^. WNT7A did not increase immunoreactive β-catenin in the blastocyst^34^ and may be acting independent of canonical pathways that are inhibitory to blastocyst formation in the cow.

An important topic for future investigation is the mechanism by which DKK1 acts on the embryo at day 5 to 7 of development to program the embryo 8 days later for trophoblast elongation. One explanation is regulation of the epigenome of the developing trophoblast. The capability of DKK1 to introduce epigenetic modifications has been observed in hepatic stellate cells where DKK1attenuates MeCP2 enrichment in the *Pparγ* promoter and H3K27me2 enrichment in exon 5 of the same gene^55^. A recent report focused on regulation of the transiently imprinted *Zdbf2* locus in the mouse has illustrated how regulation of DNA methylation in the preimplantation period can have consequences for phenotype as late as the adult period^56^. Another possibility is that DKK1 regulates lineage differentiation in the bovine blastocyst in a manner that promotes subsequent trophoblast elongation. DKK1 has been reported to increase number of trophectoderm cells in the pig blastocyst^57^. In an early paper, we reported that DKK1 increased the proportion of cells in the blastocyst that were trophectoderm or hypoblast as compared to inner cell mass^23^. We were unable to replicate these observations in a subsequent study^34^. Moreover, DKK1 decreased expression of *AMOT* in the bovine morula^30^ and use of silencing RNA genes against *AMOT* indicates that reductions in AMOT should reduce number of trophectoderm cells^58^. Therefore, the action of DKK1 on trophoectoderm cells is not consistent. Recent evidence of existence of multiple trophectoderm subpopulation in the bovine blastocyst^59^ raises the possibility that DKK1 favors differentiation of trophectoderm cells into lineages important for trophoblast elongation. DKK1 might also activate gene pathways important for trophoblast elongation. One such pathway is proposed to involve PPARG as a central regulator^18^ and DKK1 can induce expression of *PPARG* in mouse fibroblast and hepatic stellate cells^56,60,61^.

The experimental design of the study reported here involved embryo production using X sorted semen. This decision was made because sexual dimorphism has been observed in a similar context. In particular, exposure of *in vitro* produced bovine embryos to CSF2 during culture enhanced elongation and IFNT secretion for males, but have the opposite effect in female embryos on day 15 of development^46^. Although effects of DKK1 have not been different for male and female embryos in terms of development to the blastocyst stage, cell number, proportion of ICM and trophectoderm cells^34^, and transcriptome at the morula stage^30^, we limited this study to one sex in order to minimize the possibility of sex by DKK1 interactions. Females were chosen because the beneficial effect of DKK1 on pregnancy rate was observed in female embryos^23^. Also, a single sire was used for embryo production to avoid variation in embryonic size due to sire.

In conclusion, these results indicate the importance of DKK1 as an endometrial-derived regulatory molecule capable of programming development of the bovine embryo at the morula to blastocyst stage of development for subsequent trophoblast elongation. This result indicates the importance of regulation of WNT signaling for optimal development of the preimplantation embryo. Results also indicate that DKK1 is a putative progestomedin that mediates the actions of progesterone to promote trophoblast elongation in cattle. Progesterone regulates endometrial *DKK1* expression in several species and DKK1 may function as a mediator of progesterone’s action on the endometrium in multiple mammalian species. Further experiments to determine whether progesterone can regulate trophoblast elongation in females in which *DKK1* expression in the uterus is blocked would allow further examination of this hypothesis.

## Supplementary information

Supplementary Dataset 1

## Data Availability

The RNASeq data generated in the study have been deposited at Gene Expression Omnibus (GEO) with the accession number GSE126680. Other data are in the Supplementary File.
